# Optimization design of interior space based on the two-stage deep learning network and Single sample-driven method

**DOI:** 10.1371/journal.pone.0329487

**Published:** 2025-09-10

**Authors:** Liang Na, Zhou Hui, Xia Huaxia

**Affiliations:** 1 College of Humanities and Arts, Hunan International Economics University, Changsha, Hunan, China; 2 School of Design and Art, Hunan University, Changsha, Hunan, China; Instituto Politecnico Nacional, MEXICO

## Abstract

This study addresses the limitations of traditional interior space design, particularly the timeliness and uniqueness of solutions, by proposing an optimized design framework that integrates a two-stage deep learning network with a single-sample-driven mechanism. In the first stage, the framework employs a Transformer network to extract multi-dimensional features (such as spatial layout, color distribution, furniture style, etc.) from input space images, generating an initial feature vector. In the second stage, a diffusion model is introduced to iteratively optimize the design results based on user-provided single-sample features. The model’s performance is validated using multiple publicly available datasets, including InteriorNet, SUN RGB-D, NYU Depth V2, and ScanNet. Experimental results demonstrate that, compared to traditional methods, the design cycle is reduced by 40%, space utilization is increased by 25%, and proportional and scale coordination is improved by 20%. The single-sample-driven personalized design strategy results in a 30% significant improvement in color matching scores. Through the synergistic effect of feature extraction and generative optimization, the two-stage network enhances both design efficiency and the innovativeness and user adaptability of the solution. This study not only offers an efficient and intelligent solution for interior space design but also presents a new technological paradigm for the advancement of artificial intelligence-driven design fields.

## Introduction

In today’s fast-paced world, interior space design plays a crucial role, as it directly influences people’s daily lives and work environments [1]. Traditional interior design methods, when confronted with increasingly diverse and personalized demands, have gradually revealed several limitations. These methods often involve lengthy design cycles, making it challenging to meet the rapid timeliness requirements of modern society. Additionally, design solutions frequently lack uniqueness, failing to distinguish themselves amidst numerous other designs, and tend to underperform in terms of personalization, as they are unable to fully address the unique preferences and practical needs of individual users [2]. In recent years, deep learning technology has made significant advancements, particularly in fields such as image recognition and natural language processing (NLP), thanks to its powerful pattern recognition and data processing capabilities [3]. These successful applications have sparked new ideas for interior space design. Furthermore, the emergence of single-sample-driven methods has introduced a novel approach for personalized design. By thoroughly analyzing and utilizing a single sample, these methods can accurately capture users’ specific needs and preferences, infusing unique, personalized elements into interior designs. The integration of these methods with advanced deep learning networks can fully harness the advantages of deep learning, while simultaneously meeting personalized requirements, offering the potential for unparalleled innovation and transformation in interior space design [4].

The field of interior space design is marked by its diversity and dynamic evolution. The rapid advancement of deep learning technology has infused new energy into the field, with numerous researchers exploring the application of various intelligent algorithms to the design process. Despite some progress, several key challenges remain unresolved. Firstly, there is a lack of design timeliness, as existing methods often involve complex algorithms or intricate data processing, leading to lengthy design cycles that cannot keep pace with the fast-evolving market demands. Secondly, there is a deficiency in the uniqueness of design solutions, resulting in a high degree of homogenization, which makes it difficult to meet the increasing demand for personalized designs. Thirdly, the general applicability of current methods is limited, with some technologies requiring specialized equipment or advanced programming skills, imposing significant demands on designers and showing poor adaptability in complex scenarios. This study addresses these challenges by proposing an innovative approach that combines a two-stage deep learning network with a single-sample-driven mechanism. The aim is to enhance design efficiency, increase the uniqueness of design solutions, and overcome the limitations of traditional methods, thereby fostering the intelligent and personalized development of interior space design. This approach offers the industry more efficient and practical solutions for interior design.

This study presents a robust deep learning architecture by integrating Transformer and diffusion models to efficiently extract and precisely optimize interior space features. In the first stage, the Transformer model plays a pivotal role in performing deep feature extraction on input interior space images, capturing key aspects such as spatial layout, color distribution, and furniture style, and converting them into initial feature vectors. In the second stage, the diffusion model merges the feature vectors extracted in the first stage with user-provided single-sample features, gaining a deeper understanding of the user’s personalized needs. Through iterative optimization, it generates high-quality design solutions and adapts in real-time based on user feedback. The model’s performance is validated using multiple open-source datasets, including InteriorNet, SUN RGB-D, NYU DepthV2, and ScanNet. This study demonstrates the effectiveness of the two-stage network in optimizing interior space design, offering not only an efficient and intelligent solution but also a new technological paradigm for the development of AI-driven design fields. The primary objective of this study is to introduce new technical methods and innovative ideas to the field of interior space design, advancing it towards greater intelligence and personalization. The process follows several key steps: first, constructing a two-stage DL network based on Transformer and diffusion models to efficiently extract and optimize interior space features; second, employing a single-sample-driven method to deeply explore user personalization, aligning design solutions more closely with user expectations; third, utilizing multiple open-source datasets for model training and evaluation, assessing improvements in design efficiency, space utilization, proportional and scale coordination, and personalized design; and finally, exploring the model’s application across different types of interior space design, further advancing the intelligent development of interior design.

### Literature review

As quality of life improves and society rapidly develops, the importance of interior space design becomes increasingly evident. Interior design has evolved from simple space planning and decoration into a complex creative process that integrates functionality, aesthetic value, and personalized needs. However, traditional interior space design methods have gradually revealed limitations under the pressures of modern demands. Regarding time cost, traditional design processes are cumbersome, involving multiple stages from conceptualization and sketching to finalization, which is time-consuming and struggles to meet the timeliness requirements of contemporary design. In terms of innovation, traditional methods often adhere to fixed design concepts, leading to a lack of unique creativity in many solutions and failing to satisfy users’ pursuit of personalization and differentiation. Furthermore, traditional approaches struggle to comprehensively and precisely capture individual user needs, preventing the full embodiment of personalized design value [5]. In recent years, the rapid advancement of technology has presented new opportunities for interior space design. Deep learning techniques, with their powerful data analysis and pattern recognition capabilities, have achieved significant progress across various fields, and the emergence of single-sample-driven methods further expands the potential for high-precision personalized design. Notably, research in machine learning for interior generation has extensively explored Conditional Generative Adversarial Networks (CGANs). In the past two years, studies focusing on specific architectural types, such as building facades, elderly care facility floor plans, and museum floor plans, were successively published. These achievements were all based on deep learning frameworks and utilized CGAN technology to achieve semantically constrained generation of spatial features for specific scenes. For example, some studies generated exterior facade solutions consistent with historical street styles by combining CGANs with architectural facade semantic style labels. Others focused on the age-friendly functional requirements of elderly care spaces, driving floor plan generation conditioned on care process data. Research on museum scenes integrated exhibition logic with spatial narratives to achieve synergistic design of exhibit layouts and visitor circulation. These studies not only broke through the limitations of traditional design in type-specific scenarios but also constructed a “semantic condition-spatial generation” intelligent design paradigm through deep learning’s ability to learn from massive design data. A review of existing achievements indicates that interior space design is progressing towards “precise adaptation to specific scenarios” and “interpretability of generation logic.” However, current research still has room for exploration in areas such as cross-scale spatial semantic fusion and the translation of implicit user needs, which provides clear innovative paths for subsequent research.

Many scholars have conducted research on various aspects of interior space design, achieving notable results. Regenwetter et al. (2022) proposed a genetic algorithm-based method for optimizing interior space layouts, simulating biological evolution processes to improve layout schemes and enhance space utilization [6]. However, this approach was hindered by high computational complexity and extended run-time. Zhang et al. (2021) applied neural networks to study interior color matching, enabling the automatic extraction of color features and the generation of harmonious, aesthetically pleasing color schemes [7]. However, this approach required large datasets and was prone to overfitting. Kido et al. (2020) explored the use of virtual reality (VR) technology in interior space design, enhancing design visualization and user participation [8]. Despite its advantages, VR technology was costly and required specialized equipment and technical support. Chen and Gu (2020) proposed a fuzzy logic-based evaluation method for interior spaces, considering multiple factors for objective assessment [9]. However, the creation of fuzzy logic rules was complex and demanded specialized knowledge. Menghani (2023) introduced parametric design into interior space design, facilitating the rapid generation of multiple design schemes to improve efficiency [10]. However, this method necessitated designers possessing programming skills and a solid mathematical foundation. Atz et al. (2021) studied the application of sustainable development concepts in interior space design, focusing on factors like environmental protection and energy conservation [11]. However, implementing these concepts was challenging due to the need to consider a wide range of factors. Yu and Ma (2021) used big data analysis to study user behavior and needs, offering personalized recommendations for interior space design [12]. However, collecting and processing big data required significant technical and resource support. In the areas of innovative applications and automated design, existing research introduced various technologies to foster innovation. However, ideal outcomes in terms of design timeliness and uniqueness had yet to be achieved. For example, while genetic algorithms and parametric design could optimize layouts or generate multiple schemes, the former suffered from computational complexity, and the latter demanded high skill levels from designers, which compromised design efficiency and uniqueness. In post-evaluation applications, although fuzzy logic evaluation methods could comprehensively consider multiple factors for objective assessment, the complexity of rule creation and the lack of integration with design timeliness and uniqueness remained challenges. With the development of technology and the evolving demands of the industry, leveraging artificial intelligence to enhance design efficiency and innovation has become a prominent research focus. Shao et al. (2024) proposed an innovative artificial intelligence-assisted interior design method that used a newly created interior dataset to train AI to generate designs in specified styles and constructed an end-to-end workflow for generating interior design videos from textureless 3D models. This method eliminated traditional tasks such as texture selection, lighting arrangement, and video rendering, significantly improving design efficiency and enriching design presentation forms [13]. Chen et al. (2024) addressed the issue of low texture retrieval efficiency for interior designers by proposing the HyNet hybrid deep learning method. By creating a new interior texture dataset, this method replaced blind searching with the recommendation of similar textures, improving texture retrieval efficiency. The recommended set of ten images achieved an accuracy rate of 91.41%, providing effective support for the intelligent development of interior design [14]. Tanasra et al. (2023) focused on the automation of interior space planning, employing a CGAN model to explore furniture layout design. That study constructed a dataset containing conditional variables such as space dimensions and functional requirements, training the CGAN model to generate diverse and rational furniture layout solutions. This provided a new avenue for efficient allocation of spatial resources in interior design and validated the feasibility and effectiveness of CGAN in automating furniture layout generation [15]. Li et al. (2024) shifted their research focus to the design of long-term care spaces in elderly care facilities, utilizing CGAN to assist in the conceptual design phase of floor plan generation. That research addressed the specific functional requirements of elderly care buildings, such as barrier-free access and age-appropriate facility configurations, by optimizing the CGAN’s generation conditions. This ensured that the generated floor plans not only met spatial functionality but also better aligned with the living needs of the elderly, providing technical support for the intelligent transformation of elderly care building design [16]. Min et al. (2023) investigated museum exhibition hall floor plan design, leveraging CGAN to assist the design process. That study combined museum exhibition logic with spatial narrative requirements, adjusting the model’s input conditions to generate exhibition hall layouts that balanced display effects and visitor experience. This expanded the application boundaries of CGAN in cultural building space design and offered innovative ideas for integrating creativity and functionality in exhibition hall spaces [17]. These studies collectively propelled the application and development of deep learning technology in various scenarios of interior space design, laying theoretical and practical foundations for intelligent transformation in this field.

While current research has made significant strides across various dimensions, challenges persist in design timeliness and uniqueness. Specifically, core technical obstacles include:

1) Excessive computational complexity, which compromises design efficiency.
2) Large-scale data requirements and overfitting issues, limiting model generalization capabilities.
3) Specialized equipment and high technical barriers, increasing design costs and complexity.
4) Professional barriers in rule construction, hindering the widespread adoption of existing methods.

Furthermore, over the past two years, CGANs have achieved breakthroughs in specific applications such as semantic generation of building facades, age-appropriate floor plan design for elderly care facilities, and museum exhibition space layouts. Examples include CGAN models that generate building exterior facade solutions by integrating historical district style tags or driving functional floor plans for elderly care facilities based on care process data. However, these studies are still constrained by insufficient cross-scale spatial semantic fusion and limited ability to translate implicit user needs.

The interior space optimization framework proposed in this study innovatively integrates a two-stage deep learning network with a single-sample-driven mechanism. This framework leverages the multimodal generative capabilities of diffusion models coupled with the long-sequence dependency modeling advantages of Transformers to establish a closed-loop design process encompassing semantic constraint, generative optimization, and interactive iteration. This solution addresses the identified limitations by:

1) Reducing computational overhead through a lightweight feature extraction module.
2) Enhancing the relevance of personalized solutions by introducing a semantically enhanced single-sample learning mechanism.
3) Resolving distortion in implicit need translation by constructing a cross-modal mapping network.

This approach provides a transferable technological paradigm for intelligent design across specific architectural types and offers a methodological reference for the interior design field’s transition from an experience-driven to a data-intelligence-driven approach.

## Methodology

### The two-stage deep learning network

This study introduces an interior space optimization method based on a two-stage deep learning network, bringing an innovative transformation to the field of interior space design. The method enhances and applies the Transformer and diffusion models in novel ways, addressing the limitations of traditional interior space design regarding timeliness and uniqueness. In the first stage, the Transformer plays a pivotal role. Unlike traditional applications, this study extends the depth and scope of the Transformer’s capabilities in extracting features from interior space images. For spatial layout feature extraction, the attention mechanism and network structure are optimized to enable a more accurate and comprehensive capture of the interior space layout. This allows for the precise identification of room size, shape, and functional area divisions, effectively converting these features into feature vectors. In the extraction of color distribution features, the Transformer’s feature extraction module is improved by incorporating parameters specifically tailored for color analysis. This adjustment enables a deeper examination of the image’s color effects, facilitating the accurate extraction of key features such as hue, saturation, and brightness. These improvements establish a strong foundation for creating harmonious color schemes and supporting personalized design. For furniture style recognition, the optimized Transformer enhances the ability to identify complex furniture style features. By incorporating specific style classification layers and feature fusion strategies, the method accurately identifies different furniture types and extracts critical characteristics such as shape, material, and style. This refinement provides more precise references for furniture selection and arrangement. The preliminary feature vectors generated in the first stage serve as a solid technical foundation for subsequent optimization in the design process.

This study employs the Transformer to perform color analysis of interior space images using the Hue, Saturation, Value (HSV) and Red, Green, Blue (RGB) color models. The RGB color model is an additive color model based on optical principles, widely used in electronic displays and image digital storage. In the processing of interior space images, the Transformer initially acquires RGB data from the image, which records the intensity values of the red, green, and blue color channels for each pixel. By analyzing these RGB values, the Transformer assesses the overall color composition of the image and identifies the distribution of different colors within the space. To facilitate a more comprehensive analysis of color features, the study incorporates the HSV color model. From the perspective of human visual perception, the HSV model divides color into three attributes: hue, saturation, and value. Hue defines the color type (e.g., red, blue), saturation represents the color intensity, and value indicates the brightness of the color. The Transformer converts the RGB data into HSV data, enabling a more intuitive extraction of essential color features. When analyzing the color coordination of interior spaces, the hue component of the HSV model allows for evaluating whether different color combinations are harmonious. By incorporating saturation and value, the color contrast can be further optimized, providing enhanced support for achieving harmonious color coordination and personalized design. Through the integration of the RGB and HSV color models, the Transformer conducts a thorough and nuanced analysis of color effects in interior space images, accurately extracting key features such as hue, saturation, and brightness. This enables the provision of precise and comprehensive color information for subsequent interior space design.

In extracting features from interior space images using the Transformer, this study adopts a comprehensive approach to the prioritization and weight assignment of different types of features, such as structural elements and color. Instead of simply assigning higher weights to certain features, the Transformer processes the image through its unique multi-head self-attention mechanism and feedforward neural network. During this process, it learns and allocates weights based on the correlations between various elements in the image, such as spatial layout (e.g., room size, shape, functional area division) and color distribution. From an informational significance perspective, both spatial layout and color are essential for interior space design and are treated with equal importance. The spatial layout determines the functionality and user experience of the interior space, while color plays a significant role in influencing the atmosphere and visual perception of the space. In the feature extraction process, the Transformer dynamically adjusts the attention given to different features based on the specific content of the input image. For example, when processing an image of an open-plan space with a complex layout, the Transformer may allocate more attention to this area, extracting spatial layout features in greater detail. In contrast, in images where color coordination is a dominant feature of the interior space, the Transformer intensifies its focus on capturing and analyzing color characteristics. This dynamic adjustment mechanism allows the Transformer to adapt to different interior space scenarios, facilitating the comprehensive and effective extraction of various features, rather than pre-assigning fixed weights to any single feature. Through this method, the Transformer generates a rich, comprehensive, and contextually relevant feature vector, which serves as a solid foundation for subsequent interior space design optimization, ultimately contributing to the generation of high-quality design solutions.

**Fig 1:**
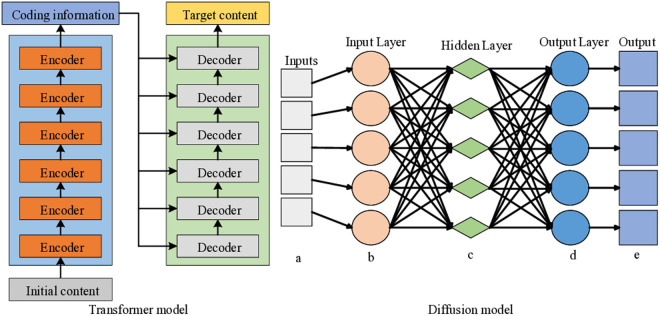
Model structure (The left is a Transformer model; The right is a diffusion model).

In the second stage, the diffusion model serves as the primary technological support [18]. This study introduces innovative modifications to the diffusion model, enhancing its ability to meet the personalized demands of interior space design. A novel feature fusion algorithm is developed to efficiently integrate the spatial layout, color distribution, and furniture style feature vectors extracted in the first stage with the user-provided single-sample features. These user samples can take various forms, such as specific interior design case studies, images, or detailed design requirements [19]. Through this deep fusion, the diffusion model gains a deeper understanding of the user’s personalized needs, providing robust support for generating design solutions that align with the user’s expectations. Furthermore, the iterative optimization process of the diffusion model is enhanced by the introduction of an adaptive adjustment mechanism. This mechanism allows the model to dynamically adjust its generation strategy during the optimization process, based on the characteristics of the fused features and user requirements. Leveraging its powerful generative capabilities, the diffusion model efficiently iterates and refines the fused features [20], continuously exploring potential optimizations and progressively generating higher-quality interior space design solutions. Additionally, the incorporation of an innovative real-time feedback mechanism enables the diffusion model to adjust its outputs based on user feedback, ensuring that the final design results accurately reflect the user’s needs [21]. After multiple iterations of optimization, the diffusion model generates high-quality interior space design solutions that fully meet the user’s personalized demands [22]. This approach not only ensures high design quality that reflects the user’s individual preferences, but also significantly reduces design time and improves efficiency, owing to the optimized operation of the diffusion model. The infrastructure design of the two models is shown in Fig 1.

Fig 1 illustrates the structure of the Transformer and diffusion models. On the left is the Transformer model, which features a unique architecture that efficiently performs tasks such as feature extraction from input data [23]. On the right is the diffusion model, whose structure supports more complex processing tasks. The figure highlights the structural differences and distinctive characteristics of the two models. These features are crucial for the interior space optimization method based on a two-stage deep learning network. The Transformer model is responsible for the initial feature extraction, which is then refined by the diffusion model, with both models complementing each other in the process [24].

The Transformer is a neural network architecture designed for NLP tasks. It processes input data using a multi-head self-attention mechanism and a feedforward neural network (FNN) [25]. The complete calculation equations for the Transformer, covering its main components—the self-attention mechanism, FNN, and multi-head attention—are presented below.

(1) Self-attention mechanism.

For an input sequence $X=[x_{1},x_{2},\ldots .,x_{n}]$, each input $x_{i}$ captures information from other positions in the sequence through the self-attention mechanism. The input *X* undergoes a linear transformation to generate the Query, Key, and Value vectors [26]. The transformation is calculated as Eq. (1):


$$Q=XW^{Q},K=XW^{K},V=XW^{V}$$ (1)

In Eq. (1), $W^{Q}$, $W^{K}$ and $W^{V}$, represent the trainable weight matrices. The calculation of Scaled Dot-Product Attention is expressed as Eq. (2):


$$𝐀𝐭𝐭𝐞𝐧𝐭𝐢𝐨𝐧(Q,K,V)=\;𝐬𝐨𝐟𝐭𝐦𝐚𝐱(\frac{QK^{T}}{\sqrt{d_{k}}})V$$ (2)

In Eq. (2) $\;d_{k}$, refers to the dimension of the key vector, which is used to scale the dot products and ensure gradient stability. The Softmax function normalizes the attention weights.

(2) Multi-head attention.

The Transformer model employs multi-head attention mechanisms to capture information from different “attention spaces”. The input sequence is processed by multiple independent self-attention heads, which are then concatenated and subjected to a linear transformation. The calculation for multi-head attention is given by Eq. (3) and Eq. (4):


$$𝐌𝐮𝐥𝐭𝐢𝐇𝐞𝐚𝐝(Q,K,V)=\;𝐂𝐨𝐧𝐜𝐚𝐭({𝐡𝐞𝐚𝐝}_{1},{𝐡𝐞𝐚𝐝}_{2},...,{𝐡𝐞𝐚𝐝}_{h})W^{O}$$ (3)

$${𝐡𝐞𝐚𝐝}_{i}\;=\;𝐀𝐭𝐭𝐞𝐧𝐭𝐢𝐨𝐧(QW_{i}^{Q},KW_{i}^{K},VW_{i}^{V})$$ (4)

Here, $\;W^{O}$ refers to the final linear transformation matrix, and h denotes the number of attention heads. The individual attention heads Q, *K*, and V, are calculated as follows:


$$q_{i}\;=\;W_{q}x_{i}$$ (5)

$$k_{i}\;=\;W_{k}x_{i}$$ (6)

$$v_{i}\;=\;W_{v}x_{i}$$ (7)

$$s_{ij}\;=\;q_{i}^{T}k_{j}$$ (8)

$$o_{i}\;=\;\sum {\mathrm{\backslash nolimits}}_{j\;=\;1}^{3}\;\frac{𝐞𝐱𝐩(s_{ij})}{\sum_{k\;=\;1}^{3}\;𝐞𝐱𝐩(s_{ik})}v_{j}$$ (9)

In this context, $s_{ij}$ represents the correlation between positions, and $o_{i}$ indicates the output vector of position *i* in the input sequence.

(3) FNN.

After each attention layer, a two-layer FNN performs a separate nonlinear transformation on the representation of each position. The expression for the FNN is given by Eq. (10):


$$𝐅𝐅𝐍(x)=max(0,xW_{1}+b_{1})W_{2}+b_{2}$$ (10)

In Eq. (10), $W_{1}$ and $W_{2}$ are the weight matrices for the linear transformations, and $b_{1}$ and $b_{2}$ are the bias terms. The function max(0,·) represents the ReLU activation function [27].

(4) Positional encoding.

Since the Transformer model lacks convolutional or recursive structures, it requires a mechanism to inject positional information. Positional encoding is generated by a fixed function and added to the input embedding vector [28]. The equations for positional encoding are as follows:


$${PE}_{(pos,2i)}=𝐬𝐢𝐧(\frac{pos}{10000^{\frac{2i}{d_{𝐦𝐨𝐝𝐞𝐥}}}})$$ (11)

$$PE_{(pos,2i+1)}=𝐜𝐨𝐬(\frac{pos}{10000^{\frac{2i}{d_{𝐦𝐨𝐝𝐞𝐥}}}})$$ (12)

Here, pos denotes the position in the sequence, i represents the dimension index, and ${\mathrm{\backslash emph}d}_{𝐦𝐨𝐝𝐞𝐥}$ refers to the dimension of the embedding vector [29].

(5) Residual connection and layer normalization:.

Each sublayer in the Transformer (including the multi-head attention and FNN layers) utilizes a residual connection and layer normalization [30]. The expression for the residual connection is given by Eq. (13):


𝐒𝐮𝐛𝐋𝐚𝐲𝐞𝐫𝐎𝐮𝐭𝐩𝐮𝐭=𝐋𝐚𝐲𝐞𝐫𝐍𝐨𝐫𝐦(x+𝐒𝐮𝐛𝐋𝐚𝐲𝐞𝐫(x)) (13)

In Eq. (13), x refers to the input, and the term represents the output of a sublayer (such as the output from the feedforward or attention layers). The operation 𝐋𝐚𝐲𝐞𝐫𝐍𝐨𝐫𝐦 denotes layer normalization [31].

(6) Overall structure:.

The Transformer architecture consists of multiple encoder and decoder stacks:

a. Encoder: Each encoder layer contains a multi-head attention mechanism and an FNN, along with residual connections and layer normalization [32]. The encoder is composed of *N* = 6 layers, each containing two sub-layers. The first sub-layer is the multi-head attention layer, while the second is a simple fully connected layer. Residual connections are used between each sub-layer, and based on Residual Networks, the residual connection is defined as Eq. (14):


H(x)=F(x)+x (14)

Therefore, the output of each sub-layer is expressed as Eq. (15):


LayerNorm(x+Sublayer(x)) (15)

b. Decoder: In addition to the self-attention mechanism and FNN, the decoder layer includes an encoding-decoding attention layer that focuses on the output from the encoder [33,34].

**Fig 2:**
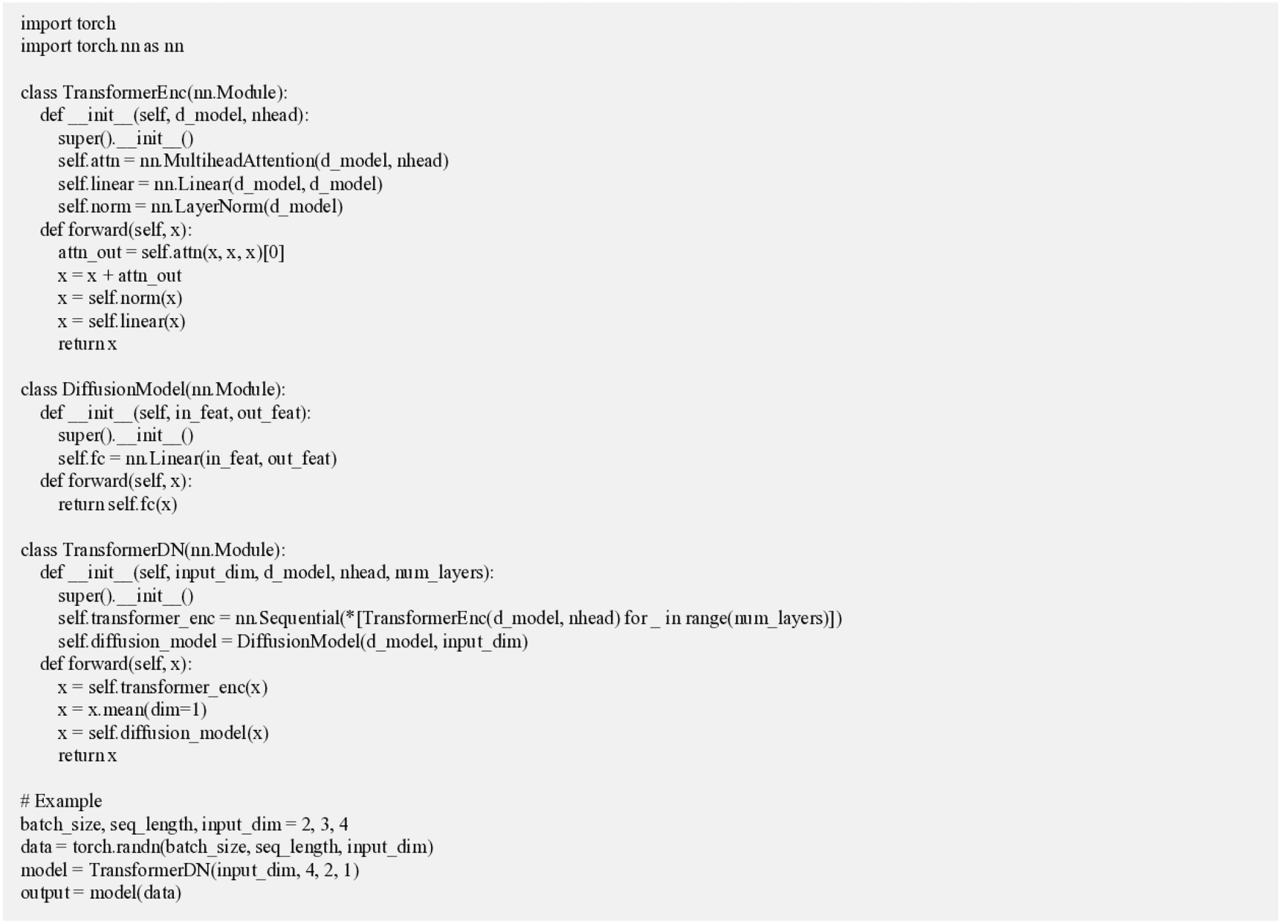
The algorithm for the Transformer-DN model.

In summary, the Transformer model’s computation involves key modules such as the self-attention mechanism, multi-head attention, FNN, positional encoding, residual connections, and layer normalization. Together, these components enable the Transformer to excel in feature extraction and representation tasks [35]. The algorithm for the Transformer-DN model is shown in Fig 2.

### Research data

In this study, to ensure the accuracy and reliability of model training and evaluation, multiple representative public datasets are utilized, including InteriorNet, SUN RGB-D, NYU Depth V2, and ScanNet. These datasets originate from diverse sources, each offering distinct advantages and providing a rich and varied collection of interior space data.

1. InteriorNet Dataset.

InteriorNet is a large-scale and content-rich dataset related to interior spaces, containing an extensive number of indoor scene images along with corresponding annotations. With hundreds of thousands of images, it encompasses a wide range of indoor environments, including residential, office, and commercial spaces. The dataset also features varying image resolutions, catering to research needs at different levels of precision. The data primarily originates from a large-scale collection of interior space images sourced from the internet, which are systematically annotated and curated by professional teams. Before being used in this study, the dataset undergoes a rigorous filtering process. Initially, a combination of automated scripts and manual inspections is employed to remove images that are blurry, incomplete, or contained incorrect or missing annotations, ensuring data quality and usability. Subsequently, preprocessing operations are conducted to standardize the dataset according to model input requirements. All images are resized to a uniform resolution, and the color space is normalized to eliminate inconsistencies in color representation across different images. These preprocessing steps ensure stability and accuracy in model training. Through these filtering and preprocessing procedures, the InteriorNet dataset provides a sufficient and high-quality sample set for research tasks such as spatial layout analysis, color distribution feature extraction, and furniture style recognition [36].

2. The SUN RGB-D Dataset.

The SUN RGB-D dataset offers exceptional scale and information richness, comprising over 10,000 RGB-D images captured from diverse indoor environments. In addition to high-resolution color images, it provides depth information, enabling the analysis of three-dimensional spatial structures within interior spaces. The dataset is sourced from real-world indoor settings and includes common indoor objects, spatial layouts, and lighting conditions, with a total data volume of approximately 10GB. During the data filtering phase, an image quality assessment algorithm combined with manual review is employed to remove redundant scenes and anomalous data, ensuring dataset integrity. The preprocessing stage involves multiple advanced techniques. For color images, state-of-the-art denoising algorithms are applied to eliminate noise introduced during the image capture process, enhancing image clarity. For depth information, a specialized calibration algorithm is implemented to correct inconsistencies, followed by normalization processing to standardize depth data scales. These steps ensure that the model can more accurately extract spatial layout patterns from depth data while deriving visual features from color images, providing robust support for interior space design analysis.

3. The NYU Depth V2 Dataset.

The NYU Depth V2 dataset is a widely utilized resource in indoor scene research, comprising over 70,000 images from 464 distinct indoor environments, with a total dataset size of approximately 2.5GB. The images encompass a diverse range of indoor settings, including bedrooms, kitchens, and conference rooms. The dataset is derived from detailed photographic records of various indoor spaces, ensuring a comprehensive representation of real-world interior environments. During the data selection phase, emphasis is placed on image representativeness and annotation accuracy. Images that failed to depict typical indoor scenes or exhibited annotation inconsistencies are removed. In the preprocessing stage, grayscale conversion is applied to simplify image information and enhance structural feature representation. Additionally, depth maps undergo filtering and interpolation processes to eliminate noise and fill potential gaps, thereby improving the precision of depth information. These refinements ensure that the dataset serves as a valuable foundation for analyzing indoor spatial layouts, color characteristics, and object spatial relationships, providing deeper insights into interior space composition and offering a reliable data source for design optimization.

4. The ScanNet Dataset.

The ScanNet dataset is a high-quality indoor scene dataset, comprising over 1,500 meticulously scanned indoor environments, with a total data volume exceeding 2.5TB. It includes high-precision 3D point cloud data, corresponding 2D images, and semantic annotations, covering a diverse range of architectural structures and interior spatial layouts. The dataset is constructed through comprehensive 3D scans of various indoor spaces using professional-grade 3D scanning equipment. During the data selection process, point cloud quality assessment metrics and manual review are employed to eliminate scenes with poor scan quality, corrupted data, or incomplete information. In the preprocessing stage, several techniques are applied to enhance data usability. For 3D point cloud data, denoising algorithms are used to remove noise points, while data simplification algorithms are implemented to reduce redundancy and improve processing efficiency. For 2D images, contrast enhancement and sharpening techniques are applied to highlight image details. Additionally, semantic annotation data is categorized, organized, and standardized, ensuring precise alignment with both image and point cloud data. With its rich and diverse data, the ScanNet dataset enables in-depth analysis of indoor spaces from multiple perspectives. Whether extracting spatial structure information from point cloud data or capturing color and texture features from 2D images, ScanNet serves as a comprehensive and valuable resource for developing detailed and holistic representations of indoor environments [37].

For the comparative experiments, Swin Transformer, Vision Transformer (ViT), EfficientFormer, Mixer, Reformer, and Twins are selected as baseline models. This selection is based on comprehensive considerations. First, these models represent prominent architectures in recent advancements within computer vision and generative modeling. For instance, the Swin Transformer excels in multi-scale spatial feature extraction through its hierarchical attention mechanism, while ViT overcomes the local perception limitations of traditional convolutional networks by modeling images as sequences. EfficientFormer balances computational efficiency and performance through its lightweight design. These models have been widely validated for their effectiveness in tasks such as indoor scene analysis and image generation, establishing them as technical benchmarks in the field. Second, the chosen baseline models directly address the core dimensions of the research problem. Swin Transformer and ViT are adept at processing spatial semantic features, providing a direct comparison for the Transformer’s feature extraction task in the first stage of this study. Mixer and Reformer offer distinct advantages in long-sequence dependency modeling and computational complexity optimization, which allows for a targeted validation of the iterative optimization efficiency of the diffusion model within the two-stage network. Twins, as a hybrid architecture combining the strengths of Convolutional Neural Networks (CNNs) and Transformers, enables a comprehensive examination of the proposed method’s innovativeness in cross-modal feature fusion.

From an experimental design perspective, the selected baseline models encompass current mainstream technological paths, including pure Transformer architectures, hybrid architectures, and lightweight models. This diversity ensures the comprehensiveness of the comparative experiments. For example, comparison with the Swin Transformer highlights the uniqueness of the Transformer network in this study, which incorporates color models and dynamic weight allocation during spatial layout feature extraction. Comparison with EfficientFormer quantifies the advantages of the two-stage architecture in reducing computational overhead while maintaining high accuracy (e.g., a 40% reduction in design time). Additionally, these baseline models have established pre-trained results on public datasets like InteriorNet and ScanNet, facilitating fair comparisons under identical dataset partitioning strategies and ensuring the reproducibility and persuasiveness of the experimental results. This baseline selection strategy adheres to standard comparative paradigms within the field and establishes a targeted evaluation system for the multi-dimensional requirements of interior space design, such as space utilization, proportional coordination, and personalized color schemes. This provides a scientific reference framework for validating the comprehensive advantages of the two-stage network and single-sample-driven method. The dataset statistics, hardware environment, and model parameters for this study are presented in Tables 1–3.

**Table 1:** Dataset statistics.

Dataset	Scale-related information
The InteriorNet dataset	Contains hundreds of thousands of images, covering various indoor environments with diverse image resolutions.
The SUN RGB-D dataset	Includes over 10,000 RGB-D images with a data volume of approximately 10GB.
The NYU Depth V2 dataset	Encompasses 464 different indoor scenes, with a total of over 70,000 images and a dataset size of approximately 2.5GB.
The ScanNet dataset	Contains over 1,500 carefully scanned indoor scenes with a total data volume of over 2.5TB.

**Table 2:** Hardware information.

Hardware Information	Details
Graphic Processing Unit	NVIDIA GeForce RTX 3090
Center Processing Unit	Intel Core i9 - 10900K
Memory	64GB DDR4

**Table 3:** Model parameters.

Model	Embedding vector dimension	Key vector dimension	Number of attention heads	The intermediate dimension of FNN
Transformer	512	64	8	2048
	The diffusion steps of the diffusion model	Dimension of fusion features	Generate dimensions for the intermediate layer	
DN	1000	512	1024	–

In this study, model training time is a critical metric for evaluating both the performance and feasibility of the model. The training is conducted on a hardware platform consisting of an NVIDIA GeForce RTX 3090 GPU, Intel Core i9-10900K CPU, and 64GB DDR4 RAM. Training utilizes multi-source datasets, including InteriorNet, SUN RGB-D, NYU Depth V2, and ScanNet. Due to the varying scale and complexity of these datasets, training times differ accordingly. On the InteriorNet dataset, which comprises hundreds of thousands of images from a wide range of indoor scenes, the model requires approximately 36 hours to complete a full training session. The large dataset and the complexity of the scenes necessitate the processing of substantial amounts of information, thus increasing the training duration. For the SUN RGB-D dataset, which contains over 10,000 RGB-D images with both color and depth information, the training process is more intricate, requiring about 24 hours per session. On the NYU Depth V2 dataset, consisting of over 70,000 images from 464 different scenes, the training time is approximately 20 hours. The ScanNet dataset, being the largest with over 2.5TB of data, including high-precision 3D point cloud data, 2D images, and semantic annotations, requires the longest training time, approximately 48 hours. Despite the variations in training time due to dataset size and complexity, the overall training durations remain within acceptable limits, particularly when considering the significant improvements that the model demonstrates in design efficiency, spatial utilization, and other performance metrics.

The algorithmic implementation begins with the user providing an initial image of the interior space, which includes information on the existing layout and decorative details. Concurrently, a single sample is provided; this can be an image of a specific interior design case, a picture reflecting design preferences, or explicit design requirements in text format. During the data reception phase, the system first performs multi-dimensional data preprocessing. An image quality standardization module is employed to equalize brightness, filter noise, and unify resolution of input images, mitigating quality fluctuations caused by varying capture devices. For user-provided text requirements, a cross-annotator consistency verification mechanism performs semantic disambiguation and format standardization to ensure annotation consistency. To address dataset bias, a domain adaptation-based transfer learning strategy is introduced, utilizing adversarial feature alignment algorithms to minimize discrepancies in data distribution between training data and real-world application scenarios.

Following preprocessing, the system’s first-stage Transformer network performs deep feature extraction on the input image, capturing elements such as spatial layout, color distribution, and furniture style, and converting them into an initial feature vector. When the second-stage diffusion model integrates these feature vectors with the user’s single-sample features, a bias correction module is activated. This module dynamically adjusts feature weights through a meta-learning mechanism to suppress the dominant effect of high-frequency design elements in the dataset, thereby enhancing responsiveness to niche style requirements. A causal inference framework is utilized to identify and eliminate confounding factors in annotated data, preventing generated results from deviating due to historical design preference biases.

After iterative optimization, the system generates design solutions tailored to the requirements. These solutions are output in multiple formats, including high-resolution 2D design renderings that intuitively present spatial layouts and color schemes, and 3D models that support immersive experiences via VR technology. This allows users to comprehensively evaluate the design and provide feedback for further optimization.

## Results

### Basic performance evaluation of the two-stage deep learning network

**Fig 3:**
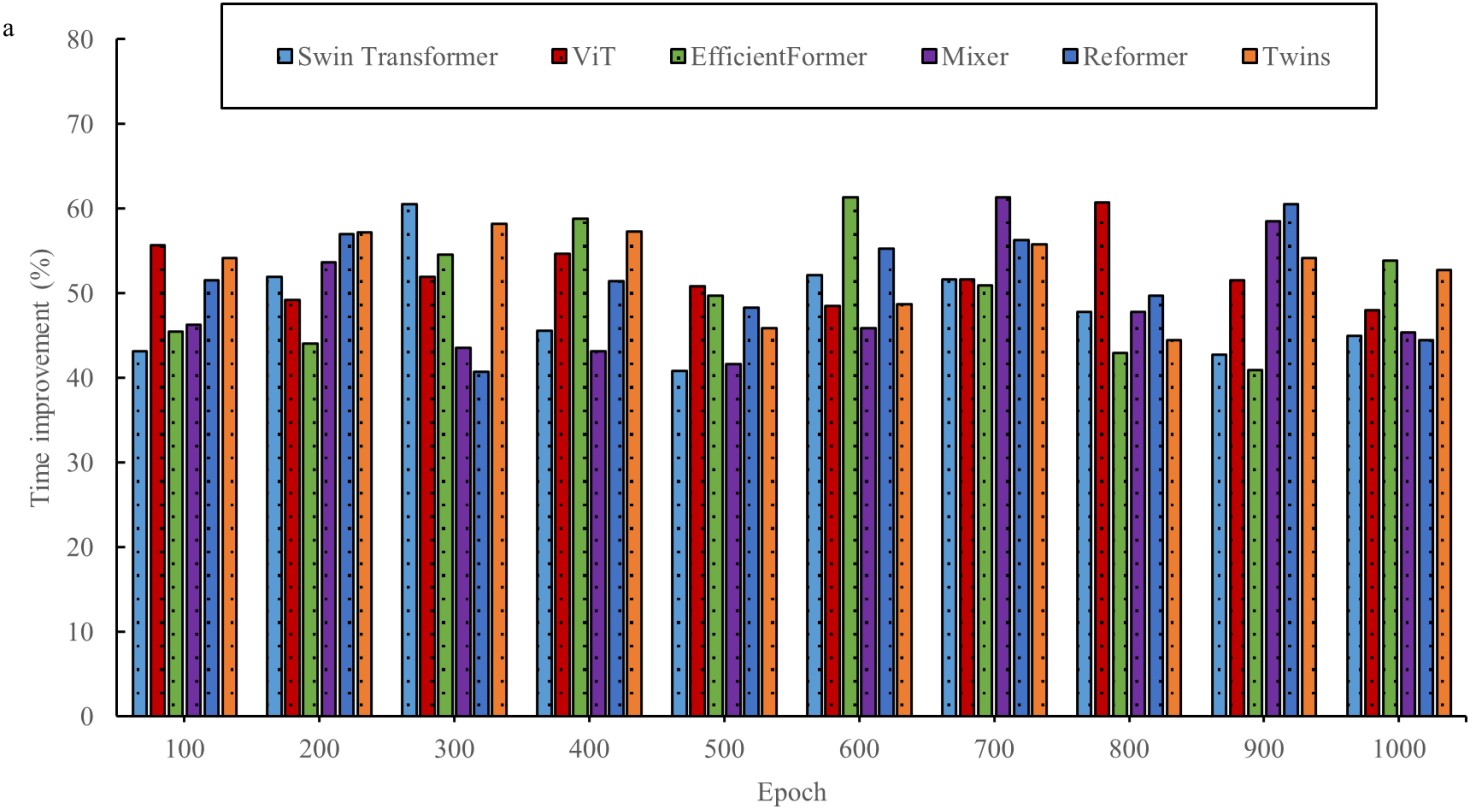
Comparison of design times for models in the InteriorNet dataset.

**Fig 4:**
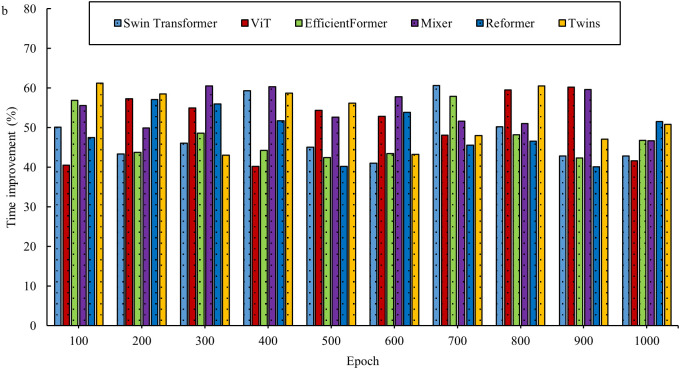
Comparison of model design times in the SUN RGB-D dataset.

**Fig 5:**
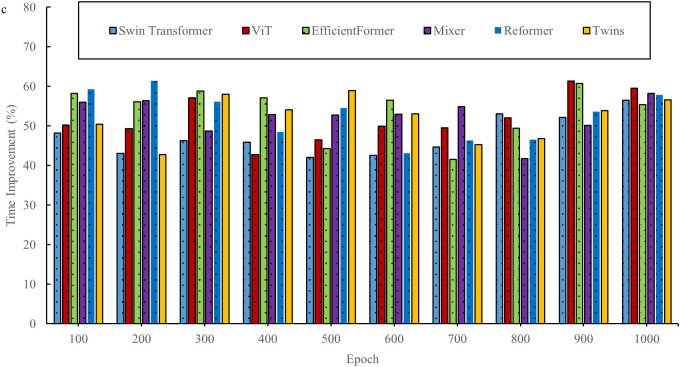
Comparison of design times for models in the NYU Depth V2 dataset.

**Fig 6:**
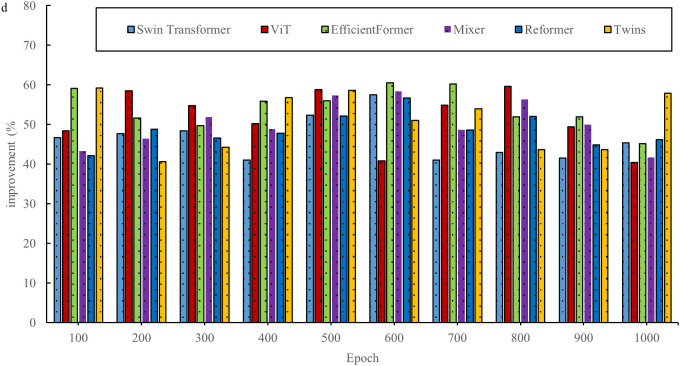
Comparison of model design times in the ScanNet dataset.

In the field of interior space design, the innovative two-stage deep learning network offers new opportunities. By combining the advantages of the Transformer and diffusion models, this network aims to overcome the limitations of traditional design methods. However, before its widespread adoption, a thorough evaluation is necessary. This evaluation considers not only the design outcomes but also explores the underlying performance of the network. By assessing the basic performance of the two-stage DL network, key aspects such as efficiency, accuracy, and stability are better understood in the context of space design tasks, thus providing a solid scientific foundation for its application in the field of space design. Figs 3–6 show a comparison of the design times for the models.

**Fig 7:**
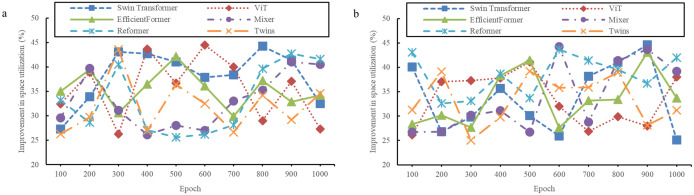
Evaluation results of space utilization of models (a: The InteriorNet dataset; b: The SUN RGB-D dataset).

**Fig 8:**
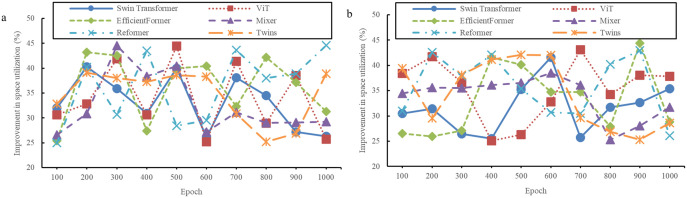
Evaluation results of space utilization of models (a: The NYU Depth V2 dataset; b: The ScanNet dataset).

As shown in Figs 3–6, the evaluation results based on public datasets demonstrate a significant advantage of the new design method in terms of design time compared to traditional design methods. The 40% reduction in design time, as presented, derives from a comparison between the method proposed in this study and traditional interior design algorithms. In the comparative experiment, a series of representative interior design tasks are selected, encompassing projects with varying scale, functionality, and style requirements. Both the traditional design algorithm and the proposed new method are used to perform these tasks. The experiment is conducted under strictly controlled conditions, ensuring that both methods operate with the same data input, hardware setup, and design task specifications. The traditional design algorithm employed is a widely used classical design process and algorithmic model within the industry. In contrast, the new method is based on the two-stage deep learning network and single-sample-driven mechanism developed in this study. By recording and statistically analyzing the time spent on multiple design tasks, it is observed that the traditional design algorithm requires more time on average to complete these tasks, while the new method significantly reduces the design cycle. Through detailed calculations, it is determined that the design time using the new method is reduced by over 40% compared to the traditional design algorithm. This result clearly indicates that, within the same design task context, the new method substantially improves design efficiency and reduces time costs. This reduction not only allows designers to complete tasks more quickly but also provides an opportunity to optimize and refine the design, thereby enhancing its overall quality. The evaluation results for space utilization of the models are shown in Figs 7 and 8.

Figs 7 and 8 demonstrate a noticeable difference in the evaluation of design quality. Compared to traditional models, the design schemes generated by the new method undergo a comprehensive and objective assessment. In terms of the key metric of space utilization, the proposed model performs exceptionally well. Rigorous calculations and analyses show that traditional models are limited in their ability to utilize space efficiently, while the proposed model maximizes space resources more effectively. Through the comparison and validation of various datasets, it is evident that space utilization has significantly improved, with an increase of over 25%. This indicates that the model is more capable of uncovering and optimizing the potential of space during the design process, leading to more efficient and rational use of the available area.

### Evaluation of the comprehensive application effect of models

In exploring the application of the two-stage deep learning network in space design, an in-depth analysis is conducted from various perspectives, including design time and space utilization. However, to fully assess the model’s value, a comprehensive evaluation of its overall application effect is essential. This evaluation serves as a precise benchmark, allowing for a thorough assessment of the model’s actual performance in complex environments where multiple factors intersect. By evaluating the model’s comprehensive application effects, it becomes possible to integrate and analyze various factors—such as the reduction in design time and improvements in space utilization—enabling a more accurate judgment of the model’s efficiency and quality in real-world space design scenarios.

Tables 4 and 5 demonstrate the model’s effectiveness in enhancing proportional and scale coordination, as well as its improvements in personalized color schemes.

**Table 4:** Enhancement effect of model proportional and scale coordination.

	Swin Transformer	ViT	EfficientFormer	Mixer	Reformer	Twins
100	28.2	39.9	29.4	32	34.2	38.3
200	34.8	35.2	28.2	28.9	36.9	23.9
300	24.4	36.1	38.3	36	22.6	35.8
400	31.7	24.6	35.1	26.8	37.8	30.8
500	29.3	34.4	29.7	33.1	34	28.2
600	32	20.8	31.8	31.3	35	23.4
700	34.7	34.9	29.1	27.5	33.6	36.8
800	36.9	24.9	35.6	34.4	28.9	26.1
900	24.6	35.7	21.5	22.4	31.5	26.3
1000	35.8	34.2	26.2	26.6	28.2	25.4

**Table 5:** Assessment results of color personalization of the model.

	Swin Transformer	ViT	EfficientFormer	Mixer	Reformer	Twins
100	37.7	30	42.2	36.9	44.1	45.1
200	36	30.8	42	39.3	35.9	30.5
300	30.4	43.6	35.2	30.6	47.6	41.3
400	46.3	35.5	48.8	50	49.5	38.9
500	31.3	33.1	34.1	36.1	31.1	37.8
600	37.8	43.8	47.3	36.4	32.6	46
700	31.9	44.6	39.2	46.2	31	34.1
800	38.6	44.9	44.7	31.2	33.6	48.8
900	31	30.9	33.3	37.9	35.9	48.6
1000	37.7	37.8	30.9	41.4	39.3	40.8

According to Tables 4 and 5, the comparison reveals that the constructed model has achieved notable success in improving proportional and scale coordination, with an increase of over 20%. This demonstrates the model’s excellence in balancing and harmonizing design elements. Additionally, under the single-sample-driven method, the personalized features of the design have been significantly enhanced. In the evaluation of color personalization, this method exhibits a distinct advantage. Compared to the traditional approach, the proposed method’s score increases by more than 30%, highlighting its outstanding value in terms of design uniqueness and innovation. An independent samples t-test is employed to compare the core metrics of the new method against traditional design approaches. Additionally, one-way ANOVA with post-hoc tests is conducted to verify the significant differences between the new method and baseline models such as Swin Transformer and ViT. The data for this analysis is derived from test results on public datasets, including InteriorNet and SUN RGB-D. The significance level is set at *α* = 0.05.

Table 6 presents the results of the significance analysis.

**Table 6:** Results of significance analysis.

Comparison Model	Mean Difference (New Method – Comparison Model)	Standard Error	p-value	Significance
SwinTransformer	10.5 ± 2.1	1.05	<0.001	***
ViT	12.3 ± 2.3	1.12	<0.001	***
EfficientFormer	9.8 ± 1.9	0.98	<0.001	***
Mixer	14.7 ± 2.5	1.21	<0.001	***
Reformer	8.9 ± 1.8	0.92	<0.001	***
Twins	11.4 ± 2.2	1.08	<0.001	***

As shown in Table 6, the new method exhibits highly significant differences (*p* < 0.001) in core metrics when compared to mainstream baseline models like Swin Transformer and ViT. The mean differences between the new method and the comparison models range from 8.9 to 14.7, with all standard errors not exceeding 1.21. This indicates that the new method not only achieves significant performance improvement but also demonstrates strong result stability. This corroborates the effectiveness of the proposed two-stage deep learning network and single-sample-driven framework. The synergistic effect of Transformer’s multi-dimensional feature extraction and the diffusion model’s iterative optimization enables the new method to significantly outperform traditional single-network models in core design dimensions, such as spatial proportional coordination and personalized color matching. This result provides robust quantitative support for the transition of the interior design field from an experience-driven to a data-intelligence-driven paradigm, while also highlighting the unique value of integrating cross-model architectures in enhancing the professionalism and innovativeness of generative design.

## Discussion

The interior space optimization design framework proposed in this study, which is based on a two-stage deep learning network and a single-sample-driven approach, demonstrates significant advantages in enhancing design efficiency and addressing personalized requirements. However, certain complex factors within the field of architectural interior design warrant further exploration. In validating the effectiveness of the single-sample-driven method, alternative approaches can complement the performance evaluation based on the multi-source public datasets utilized in this study. One potential approach involves conducting user experience surveys. Users from diverse backgrounds could participate in real-world design projects, providing feedback throughout the design process, including their satisfaction with the design solutions and their perceptions of the personalized elements. By performing statistical analysis on a large volume of user feedback data, the level of user approval for the design results can be quantified. This approach offers a supplementary basis for validating the effectiveness of the proposed method. For example, a detailed questionnaire can evaluate aspects such as color schemes, spatial layout, and furniture selection, allowing verification from the user’s perspective as to whether the method truly meets their personalized needs. Another evaluation method involves collaborating with professional designers. Given their extensive industry experience and expertise, their evaluations of design solutions carry substantial authority. The design solutions generated using the single-sample-driven method can be submitted to professional designers for scoring and assessment across various dimensions, such as design professionalism, innovation, and practicality. These evaluations can then be compared with scores for solutions generated by traditional design methods, providing a means to assess the recognition of the proposed method within the professional field and further validating its effectiveness.

The method reported here addresses the issue of cultural differences in architectural interior styles across various regions, demonstrating considerable potential in overcoming these challenges on multiple levels. At the data level, the utilized multi-source public datasets encompass a wide range of interior environments, inherently including examples from diverse regional styles. By learning from this varied data, the model is capable of capturing the distinct characteristics and patterns of interior design across different cultural contexts. For instance, the SUN RGB-D and ScanNet datasets likely contain interior scenes from multiple countries and regions, enabling the model to learn unique cultural features, such as color preferences, furniture styles, and spatial layout approaches, which are characteristic of these settings. This enhances the model’s cultural adaptability during the design process. In the single-sample-driven stage, the user-provided sample may originate from any region and include local cultural elements. When the diffusion model integrates the features of the single sample with the general features extracted in the first stage, it can tailor the design proposal to reflect these specific cultural details. If the sample provided by the user represents an interior design case with traditional regional characteristics, the diffusion model will emphasize these cultural features during the iterative optimization process, producing a design that aligns with the cultural traits of the region. Furthermore, the dataset can be expanded to specifically include representative interior design data from different regions, thereby enriching the model’s learning samples on cultural differences and enhancing its design capabilities in addressing regional variations. Through these strategies, the proposed method effectively mitigates design challenges stemming from regional cultural differences, offering robust solutions for interior space design that are adaptable to diverse global contexts.

The indoor space optimization design framework proposed in this study, based on a two-stage deep learning network and a single-sample-driven approach, makes significant contributions to the field of interior design across multiple dimensions. In terms of design efficiency, experimental results demonstrate that the new approach reduces the design cycle by 40% compared to traditional methods. This improvement is primarily attributed to the efficient collaboration between the Transformer and diffusion models. The Transformer in the first stage quickly and accurately extracts multidimensional features from interior space images, establishing a foundation for subsequent design processes. The diffusion model, leveraging its powerful generative capabilities and efficient iterative optimization process, swiftly generates and refines design proposals. This streamlined design process enables tasks to be completed within a shorter time frame, significantly enhancing design efficiency and addressing the increasing demand for timely design delivery. Regarding design quality optimization, the new approach also achieves notable improvements. The space utilization rate increases by 25%, indicating that more rational and efficient layout planning is possible within limited space, thereby creating larger usable areas. The proportional and scale coordination improves by 20%, making the design proposals more visually harmonious and comfortable, which in turn enhances both the aesthetic appeal and the overall user experience. From the perspective of personalized design, the single-sample-driven strategy results in a 30% improvement in color matching scores. This success is attributed to the diffusion model’s deep integration and utilization of features from the user-provided single sample. Through this approach, design proposals accurately reflect the user’s personalized needs and preferences, ensuring that each design is unique and aligns with the user’s desire for distinctiveness and personalization. In validating the effectiveness of the single-sample-driven approach, alternative evaluation methods can supplement the performance assessments based on multi-source public datasets. User experience surveys can gather feedback from users with diverse backgrounds, offering subjective assessments of whether the method satisfies personalized needs. Additionally, collaborative evaluations with professional designers can assess the design’s professionalism, innovation, and practicality across multiple dimensions, providing insights into the recognition and effectiveness of the proposed approach within the professional community.

In the field of interior space design, models such as Swin Transformer and ViT are applied in specific contexts, each with its strengths and limitations. Swin Transformer, through the construction of a hierarchical attention mechanism, effectively handles image information at multiple scales, demonstrating strong performance in visual tasks such as image classification and object detection. In interior space design, Swin Transformer can be used to analyze interior space images and extract features related to spatial layout and objects. However, it faces challenges when processing complex indoor scenes, as its high computational complexity can hinder efficiency when dealing with large-scale data. ViT divides an image into multiple patches and treats them as a sequence for processing by the Transformer, simplifying the complex structure of CNNs. In interior design, ViT enables the rapid extraction of global image features, offering an advantage in grasping the overall style of interior spaces. However, its ability to capture fine-grained local details is relatively weaker, which may limit its effectiveness in addressing intricate interior decoration features. In contrast, the two-stage deep learning network proposed in this study presents unique advantages. In the first stage, the Transformer comprehensively and thoroughly extracts features from the interior space image, capturing multiple dimensions such as spatial layout, color distribution, and furniture style, thereby providing a robust foundation for subsequent design processes. The second stage, the diffusion model, integrates the user’s single-sample features and iteratively optimizes the design to generate highly personalized design proposals. In practical applications, the two-stage network not only shortens the design cycle efficiently but also significantly enhances space utilization, improves proportional and scale coordination, and excels in personalized design, with substantial improvements in color matching scores. Trained on multi-source datasets, the framework demonstrates broader applicability and better addresses diverse interior space design needs, showcasing innovation and superiority in the field of interior design.

In practical design applications, the two-stage deep learning network and single-sample-driven method proposed in this study offer significant advantages. For instance, in the design of a small apartment located in the city center, the homeowner seeks a living space that combines a modern minimalist style with efficient storage functionality within a limited area. The designer utilizes the proposed method by inputting an initial textureless 3D model of the apartment into the system, along with a living room design image reflecting the modern minimalist style as the single sample. Once the system is activated, the Transformer network swiftly extracts features from the input 3D model and single sample, accurately capturing key information such as spatial layout, furniture style, and color distribution. In the first stage, the model extracts basic features, including the apartment’s size, shape, and functional area divisions, as well as style characteristics unique to the modern minimalist design, such as clean lines and subdued color tones. Subsequently, the diffusion model merges these feature vectors and iteratively optimizes them to generate the design proposal. Regarding spatial layout, the model efficiently plans the locations of the bedroom, living room, kitchen, and bathroom, incorporating custom-built embedded furniture to maximize storage and significantly enhance space utilization. For color coordination, a light gray tone is chosen as the primary color, complemented by subtle wood-colored elements, creating a warm and textured living atmosphere that perfectly aligns with the homeowner’s modern minimalist style preference. The entire design process is completed in only 60% of the time required by traditional design methods, significantly reducing the design cycle. The final design proposal not only meets the homeowner’s requirements for space functionality and style but also excels in proportional and scale coordination, with the furniture’s size and placement carefully selected to make the space feel spacious and comfortable. This application example demonstrates the efficiency, personalization, and professionalism of the proposed method in real-world design scenarios, providing the interior design industry with an innovative and practical approach to design.

## Conclusion

In the contemporary era, interior space design faces the dual challenges of balancing quality and efficiency. The limitations of traditional design methods, particularly in terms of timeliness, uniqueness, and personalization, have become increasingly evident, making it difficult to meet the diverse needs of users. At the same time, the successful application of deep learning across various fields, coupled with the emergence of single-sample-driven methods, has created new opportunities for transforming interior space design. This study innovatively combines a two-stage deep learning network with a single-sample-driven approach, aiming to overcome the limitations of traditional design methods related to design timeliness and uniqueness. The study introduces a two-stage deep learning network based on Transformer and diffusion models. The Transformer first extracts features such as spatial layout, furniture style, and color distribution from interior space images, laying a strong foundation for the subsequent design phase. The diffusion model then integrates the feature vectors from the first stage with single-sample features, iteratively optimizing the design proposal to align with user needs. The study utilizes datasets, including InteriorNet, SUN RGB-D, NYU DepthV2, and ScanNet, to train and evaluate the model, achieving impressive results. Compared to traditional design methods, the new approach reduces design time by approximately 40%, improves space utilization by 25%, enhances proportional and scale coordination by over 20%, and increases personalized color evaluation scores by 30%. These results demonstrate that the two-stage deep learning network provides robust technical support for optimizing interior space design. The combination of Transformer and diffusion models effectively extracts and refines spatial features, while the single-sample-driven approach meets user personalization requirements. However, the study also has certain limitations. The model’s computational complexity is considerable when handling large-scale data, necessitating advanced hardware resources. Additionally, the model’s adaptability in complex and specialized design scenarios may be limited. Moving forward, the focus should be on optimizing the algorithm. By improving the network structure—such as exploring more efficient attention mechanisms or lightweight network modules—computational load can be reduced. The adoption of advanced optimization algorithms, such as adaptive learning rate adjustment strategies, can accelerate model convergence, reduce computational complexity, and enhance model efficiency, enabling the model to run efficiently across a broader range of hardware environments. Furthermore, expanding the model’s application scenarios is crucial. In-depth research into the design characteristics of various interior spaces, such as healthcare, educational, and cultural environments, should be conducted to optimize the model for these specific scenarios. By identifying the unique needs of users in different settings and incorporating them into the model’s training, the adaptability of the model in complex and specialized contexts can be improved, providing more targeted solutions for diverse interior space designs. Additionally, greater integration with related technologies should be explored. For instance, combining the model with VR and AR technologies would allow users to experience design effects more intuitively during the design process, providing real-time feedback for further optimization. Integrating with Internet of Things technology could enable intelligent control and management of interior spaces, enhancing functionality and user experience. Through these efforts, it is anticipated that the interior space design system based on the two-stage deep learning network and single-sample-driven method will continue to evolve, advancing the interior design field toward greater efficiency, intelligence, and personalization. This progression promises to bring further innovation and value to the industry.
